# A Workflow for Studying the Stump–Socket Interface in Persons with Transtibial Amputation through 3D Thermographic Mapping

**DOI:** 10.3390/s23115035

**Published:** 2023-05-24

**Authors:** Andrea Giovanni Cutti, Federico Morosato, Cosimo Gentile, Francesca Gariboldi, Giovanni Hamoui, Maria Grazia Santi, Gregorio Teti, Emanuele Gruppioni

**Affiliations:** 1 Centro Protesi Inail, Via Rabuina 14, Vigorso di Budrio, 40054 Bologna, Italy; f.morosato@inail.it (F.M.); c.gentile@inail.it (C.G.); g.hamoui@inail.it (G.H.); gr.teti@inail.it (G.T.);; 2 Department of Industrial Engineering, University of Padova, Via VIII Febbraio, 2, 35122 Padova, Italy; francesca.gariboldi@phd.unipd.it (F.G.); mariagrazia.santi.97@gmail.com (M.G.S.)

**Keywords:** 3D thermography, thermal imaging, lower limb amputation

## Abstract

The design and fitting of prosthetic sockets can significantly affect the acceptance of an artificial limb by persons with lower limb amputations. Clinical fitting is typically an iterative process, which requires patients’ feedback and professional assessment. When feedback is unreliable due to the patient’s physical or psychological conditions, quantitative measures can support decision-making. Specifically, monitoring the skin temperature of the residual limb can provide valuable information regarding unwanted mechanical stresses and reduced vascularization, which can lead to inflammation, skin sores and ulcerations. Multiple 2D images to examine a real-life 3D limb can be cumbersome and might only offer a partial assessment of critical areas. To overcome these issues, we developed a workflow for integrating thermographic information on the 3D scan of a residual limb, with intrinsic reconstruction quality measures. Specifically, workflow allows us to calculate a 3D thermal map of the skin of the stump at rest and after walking, and summarize this information with a single 3D differential map. The workflow was tested on a person with transtibial amputation, with a reconstruction accuracy lower than 3 mm, which is adequate for socket adaptation. We expect the workflow to improve socket acceptance and patients’ quality of life.

## 1. Introduction

After lower limb loss, individuals with amputation are involved in a prosthetic rehabilitation treatment which aims at maximizing the recovery of the lost functionality [1]. The return to an active social life is largely dependent on the artificial limb, typically consisting of mass-produced components, such as a prosthetic foot and/or knee, and a custom-made socket [2,3].

The socket, with the possible interposition of a liner, is the interface between the residual limb and the prosthetic device. For this reason, a properly shaped and built socket must promote soft tissues’ integrity and allow the stump to act as the motor and controller of the artificial limb during daily life activities, by managing the load transfer between the body and device. These are key factors for determining the success of the prosthesis [4]. A defective socket fit leads to poor biomechanical performances, discomfort, pain, inflammation and skin lesions [5]. All these factors may contribute to reduced satisfaction with the prosthesis, a lower quality of life (QoL) and the potentially abandonment of the device [6]. In [7] the QoL of individuals with unilateral transfemoral amputation was studied using the SF-36 questionnaire. The most frequently reported problems associated with a reduced quality of life were heat/sweating in the prosthetic socket (72%) and sores/skin irritation from the socket (62%), factors that can cause the local inflammation of the skin and may lead to pain and suffering [8].

The fitting of a prosthetic socket is an iterative process of trial and modification to optimize the socket volume and shape [9]. The process typically requires the user’s feedback when donning the socket, standing and walking, and the assessment of the socket position and motion relative to the residual limb by a certified prosthetist, possibly with the support of qualitative clinical measurements [10,11,12]. When one of these elements is unreliable or missing and cannot be compensated by the others, reaching a successful fitting can be an inefficient and stressful experience, with uncertain outcomes. This can be particularly critical for first-time prosthetic users, who are unaccustomed to providing specific feedback, or when the persons with amputation have a reduced, partial or absent sensation in the residual limb or are overwhelmed by allodynia, hyperalgesia, phantom pain or psychological distress.

When this is the case, objective measures can support decision-making. Among them, the assessment of temperature variation on the residual limb skin may provide valuable information for certified prosthetists about the user’s comfort with a newly developed socket which can, potentially, improve the optimization of the socket quality and performance.

Thermography allows us to measure the temperature pattern over the external surface of an object and has been already adopted in industrial [13] and biomedical applications [14]. For instance, temperature variation has been used to monitor patients with diabetes for the early detection of neuropathy and vascular disorders, such as diabetic foot disease [15]. Some studies have analyzed the use of 2D thermography in the prosthetic field [16]. The first quantitative study performed in 1984 [17] demonstrated that thermography can be used to detect phantom or residual limb pain, which is often associated with typical thermal patterns. Specifically, circulation disturbance was associated with distinct lower temperatures in the distal portion of the residual limb, while an asymmetrical increase in temperature was found in areas corresponding to pressure points, infection or locally painful spots.

In [18] the residual limb skin temperature during prosthesis use was monitored. The results showed that temperature variations over the limb surface were associated with prosthetic socket material and subject activity. In [19], the authors investigated the application of 2D thermography to assess the stump–socket interface in transtibial prosthesis users by comparing the thermal maps induced by the mechanical stresses on the limb. By using thermography and temperature sensors fitted in the socket, five hot spots were identified before and after walking. In [20], the biomechanical and thermographic analysis of the transtibial limb–socket interface was investigated. The study demonstrated that the use of thermal imaging for detecting pressure points in the socket is a suitable method for rapidly assessing the quality of the socket during its manufacturing process. In [21], 16 subjects with transfemoral amputation were monitored through 2D thermography to investigate the effect of prosthesis misalignment on the skin temperature of the limb. The results showed that prosthesis misalignment induced a variation in the temperature distribution on the limb and that, therefore, thermography can be adopted to refine the prosthesis alignment.

In these studies, which represent the available literature about the application of thermography in the clinical practice with persons with lower limb amputation, the main limitation is represented by the intrinsic bi-dimensional nature of the thermal information acquired with thermal cameras. In fact, due to the variable, subject-specific conformation of the residual limb, it can be difficult to have a full and accurate understanding of the extension and location of the hot/cold spots on the real-life limb, and then proceed with localized socket adjustments. In addition, it can be cumbersome to examine several separated 2D views to examine the full residual limb shape, particularly when comparing different conditions (e.g., rest vs. walking).

This highlights the need for a 3D solution to integrate morphological and thermal information. Some authors investigated the combination of 2D thermal images and CT medical images to map temperature information on 3D anatomical targets and improve clinical diagnosis [22], or to solve clinical issues related to the diabetic foot. In [23], 2D thermal information was used as a feature to train a neural network for the segmentation of the diabetic foot. An algorithm for the 3D registration of 2D thermal maps on 3D reconstructions of diabetic feet was proposed in [24]. The algorithm was face-validated, but no information about registration accuracy was provided. As the registration error is considerably affected by details of the volume object reconstruction [25], algorithms based on high-resolution CT images were proposed. However, despite the high accuracy of CT images, which help improve registration performances, such an approach is not practical in clinical practice due to ethical, monetary and logistical burdens, especially for socket manufacturing.

Alternative approaches were reported in the literature to collect the 3D morphology with digital scanners or photogrammetry, in combination with thermal cameras, either in stationary or handheld systems [26]. However, there is a lack of suitable protocol for subjects with lower limb amputations, (i) with the intrinsic capability of monitoring the 2D (thermal)-3D (spatial) registration error, (ii) and based on mostly freely available software (therefore, easy to reproduce in clinical centers).

The aim of this study was to address these open issues by proposing an original workflow to calculate 3D thermographic maps of the residual limb of transtibial amputees for monitoring the temperature variations induced by prosthesis use. The workflow comprises a data acquisition and a data processing technique, whose application is demonstrated in a representative case study, including a person with transtibial amputation.

## 2. Materials and Methods

First, the measurement instruments and 3D thermographic markers are described. Second, the workflow is presented to provide an overview of our approach. Third, we focus on data collection for thermal images, 3D surfaces and thermal camera optics calibration. Fourth, we report on the data processing technique, which is formed by three parts, namely the integration of the 2D set of thermal images and the 3D model, the calculation of the 3D differential thermographic map and the assessment of the process accuracy. Finally, the materials and methods relevant to the exemplary case study are reported.

### 2.1. Measurement Instruments and Rotating Platform

For the acquisition of the 2D thermal images, a commercial infrared camera FLIR A325 (Wilsonville, OR, USA) was used, with the possibility to export data in a table format. The sensor characteristics are reported in Table 1. The FLIR proprietary software was used for real-time visualization and for the setup of the acquisition parameters. Specifically, the ambient parameters affecting infrared temperature estimation (ambient temperature, humidity) were measured through an Amprobe TR300 (Everett, WA, USA). The human skin emissivity coefficient was set to 0.98 [27,28].

The FLIR camera was mounted on a tripod, at a fixed distance of 120 cm from the central axis of a squared rotating platform, with the focal plane parallel to the platform’s side [19]. The platform was surrounded by parallel bars on three sides, which were specifically designed to host the patient in an upright posture with the residual limb positioned vertically over the platform axis of rotation. By spinning the platform, it is possible to collect images in different views with no need for the patient to move voluntarily or for camera adjustments.

For the acquisition of the 3D mesh of the residual limb, a Microsoft Kinect (Microsoft, Redmond, WA, USA) was adopted in combination with the Skanect software (Occipital Inc., Boulder, CO, USA). The accuracy and resolution of the Kinect system as a 3D scanner has been already documented elsewhere [29], and the device has a sampling frequency of 20 fps.

### 2.2. 3D Thermographic Markers

**Figure 1:**
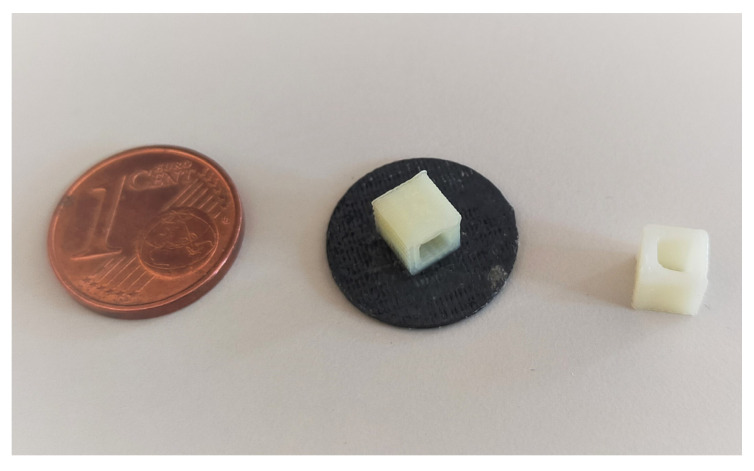
3D thermographic markers adopted in the study.

3D thermographic markers (3DTM) were conceived to facilitate and access the quality of the spatial registration of the thermal images on the 3D mesh. The 3DTM were developed to be small objects of known geometry that could be visible both in the 3D mesh and in the thermal image, and that could be positioned on the subject’s body without obstructing clinically relevant hot/cold spots on the residual limb [19,21]. To fulfill these constraints, we 3D-printed small hollow cubes in ABS, with only 4 lateral faces, with an edge length of 5 mm with a 0.5 mm think flat base of 14 mm in diameter (Figure 1).

A preliminary test confirmed that markers of this size can be handled with confidence and that the ambient air passing through the cube ensures its visibility in the thermal image. Moreover, markers are detected by Kinect and are visible in the 3D mesh. 

### 2.3. Workflow

**Figure 2:**
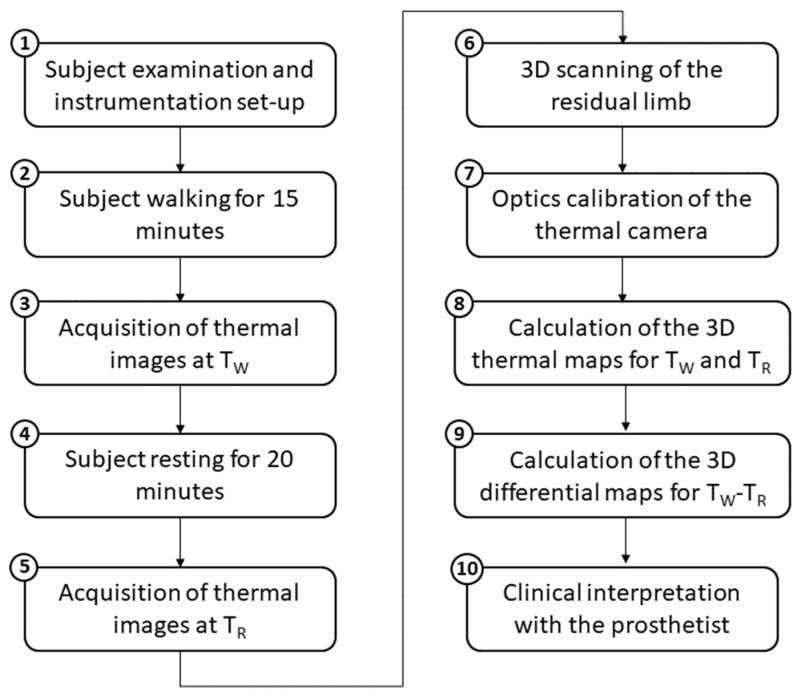
The 10-phases workflow for clinical assessment of the thermal pattern on the residual limb.

The workflow was developed to collect the skin temperature of the residual limb of a person with transtibial amputation under two conditions, to superimpose the two temperatures dataset on a single 3D model of the residual limb and to calculate a 3D differential thermal map to highlight the temperature variations between the two conditions. For instance, the two conditions can be assumed to be the skin temperature after walking (TW) and at rest (TR). The 10-phases workflow is illustrated in Figure 2. The steps are described in detail in the next sections.

### 2.4. Subject Examination and Collection of the Thermal Images

We report here about the details for phases 1 to 5 of the workflow.

Phase 1: the patient is invited to doff the prosthesis and the residual limb is examined by the prosthetist to identify areas of clinical interest where 3DTM should not be positioned. The patient is invited to familiarize themself with the rotating platform, taking advantage of the parallel bars for stability. The height of the camera tripod is adjusted to center the image plane at the middle of the residual limb. The views for image collection are established: at least 7 images are required, starting from a frontal plane roughly in steps of 45° around the limb long axis; the addition of an image of the distal end of the residual limb might be needed for some residual limbs, depending on their shape. Once the views are decided, the position of the 3DTM is set to ensure that at least three are visible in each view without obstructing the clinically relevant areas discussed with the prosthetist.

Phase 2: the patient is asked to walk with the prosthesis for 15 min as they desire, completing the activity in the measurement laboratory by sitting on a chair positioned on the rotating platform. 

**Figure 3:**
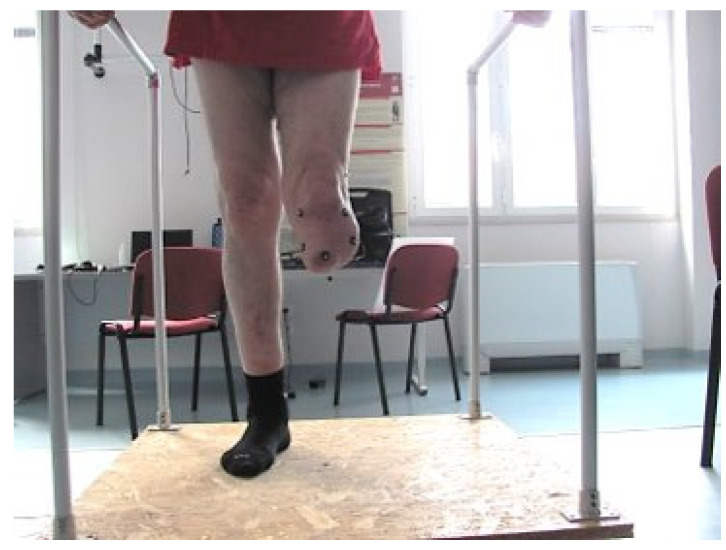
Subject with left transtibial amputation standing upright on the rotating platform with markers placed on his residual limb.

Phase 3: the patient doffs the prosthesis and 3DTM are quickly positioned on the residual limb. Then, the patient stands up with the residual limb positioned vertically over the platform rotation axis and thermal images are collected based on the pre-determined views, by adding additional images if deemed clinically important by viewing the thermal images in real-time. If the residual limb end is collected, the subject is asked to flex the knee. For medial views, the subject is asked to flex the hip to ensure good visibility (Figure 3).

Phase 4: the subject rests comfortably for 20 min in the laboratory room, while the ambient temperature and humidity are monitored. 

Phase 5: thermal images are collected again, without removing the 3DTM.

### 2.5. Data Acquisition: 3D Surface of the Residual Limb

Phase 6: the digitalization of the residual limb surface takes place after thermal image acquisition, without removing the markers or asking the subject to step down from the platform. The Kinect is hand-held and manually moved around the residual limb thanks to its small size [66 × 249 × 67 mm] and limited weight [1.4 kg]. During scanning, the operator is guided on the body parts yet to be digitalized by the real-time visual feedback of the reconstructed surface. For data collection on the residual limb, Skanect was set to “body mode” and “high” feedback quality. For optimal accuracy, all scans were completed at less than 1 m from the target. For cleaning and cropping the 3D mesh to the residual limb, the software MeshLab (ISTI-CNR, Pisa, Italy) was used. 

### 2.6. Optics Calibration of the Thermal Camera

Phase 7: the calibration of the optics of the thermal camera is completed at the end of the measurement sessions. If the camera distance to the axis of rotation of the platform and the orientation of the image plane remain as prescribed (120 cm and parallel to the platform, respectively), there is no need to repeat the camera calibration at every patient assessment.

**Figure 4:**
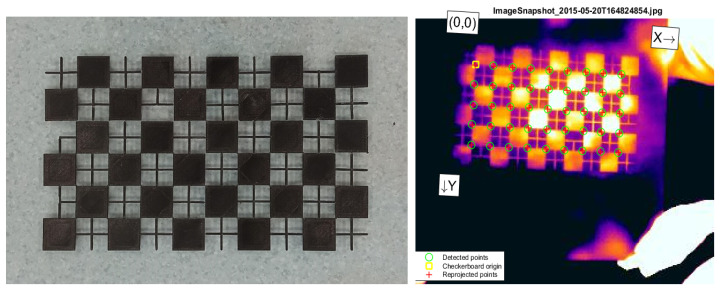
Chessboards for the calibration of the thermal camera as it appears in visible and infrared.

The Matlab Camera Calibration App (Mathworks, Natick, MA, USA) is used to calibrate the optics of the thermal camera. A rectangular chessboard composed of 30 × 30 mm black squares interconnected by thin crosses were 3D-printed in ABS to generate a bi-dimensional calibration pattern (Figure 4, left). The chessboard has a size of 300 × 180 mm, with a thickness of 2 mm and was glued on a clear, rigid PETG board, which is easy to find in prosthetic and orthotic facilities. Once heated by hand rubbing, the ABS chessboard becomes clearly visible in the thermal images (Figure 4, right).

Calibration consisted of collecting 20 images, each depicting the chessboard in a different orientation, but with a maximum inclination of 45° relative to the image plane. The focus was never changed during calibration. The calibration was considered acceptable if the reprojection error provided by the App was lower than 1 pixel.

### 2.7. Data Processing: Integration of a 2D Set of Thermal Images and 3D Model

**Figure 5:**
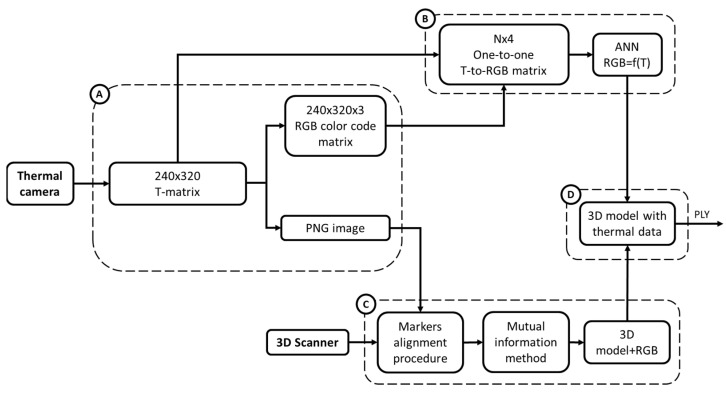
Flowchart for the integration of the thermal data into the 3D limb model. (T = temperature, N = number of samples). The flowchart is subdivided in 4 branches (A–D) each addressing a specific set of operations on the data collected by the thermal camera and the 3D scanner, to finally obtain a ply file with the 3D model incorporating thermal data.

Phase 8: To integrate the thermal data into the 3D limb model, a process combining Matlab and MeshLab was developed based on the flowchart presented in Figure 5, which consists of 4 branches, from A to D.

In branch A, the thermographic data of each thermal image were exported from the thermal camera and then imported in Matlab as a raster 240 × 320 pixel matrix containing the temperature values (Figure 5, Box A), herein referred to as the temperature matrix.

The MATLAB’s Image Processing Toolbox was used to generate a color-coded image in a PNG format of each 240 × 320 temperature matrix (Matlab jet colormap) and the corresponding 240 × 320 × 3 RGB matrix, with lower and upper color saturation anchored to 28° and 37° degrees, respectively.

Once this step was completed, the process split in two branches, namely B and C, to then re-converge into the final branch D to generate the 3D thermographic map.

The first step of branch B consisted of reading each element of the temperature matrix and the associated RBG tripled to generating an association table between the temperature values and RGB codes. The second step consisted of ordering the table in ascending temperature values and removing duplicates. When multiple temperatures differing by cents of degrees were associated with the same RBG code, a single temperature value was calculated as the average of these temperatures and associated in the table with the RGB code. Therefore, at the end of this step, a 1:1 table was obtained, in which unique temperature values were associated with unique RBG codes. As a third step, half of the table rows were randomly selected and used to train an artificial neural network (ANN) in MATLAB (Radial Basis Networks newrb), to estimate the RGB code for temperature values not in the table. The other half of the rows were used to test the ANN predictions. Hidden nodes were added until a mean squared error of 10^−4^ was reached.

The first step of branch C consisted of importing the PNG images and the 3D mesh in MeshLab for texturing. To achieve this aim, each PNG image was aligned with the 3D model in its correct position and orientation, using a two-step process. Firstly, the 3DTM positioned on the residual limb, visible in both the raster images and the 3D mesh model, was used as a reference point for initial manual alignment. Then the image-to-geometry registration procedure described by [30] was exploited, which is based on a mutual information method. Then, to project images on the 3D model, the MeshLab function parametrization and texturing from the raster was used, which requires a limited set of images (10 at most). Each portion of the surface is assigned to the “best” image based on various matrices, and the texture mapping helps preserve the fine color details [31]. The texture was transferred to the vertices of the 3D model, which was saved as a PLY file with color attributes to its vertex.

In the final branch D, the ANN from branch B and the 3D model with color vertices from branch C were imported in MATLAB. For each vertex, the RGB code was converted in a temperature value and a new PLY 3D model with temperature values for each vertex was saved. This step is required because in branch C, new colors might be generated due to the smoothing of transitions, as defined by [30]. These colors might not have a one-to-one correspondence in the temperature-to-RBG matrix available at the beginning of branch B. 

### 2.8. Data Processing: Calculation of the Differential 3D Map

Phase 9: Given a subject, the workflow can be applied in images collected under changing conditions, e.g., to assess differences in skin temperature between rest and after walking. Since the temperature information is recorded at vertex level, it is straightforward to calculate, for each vertex, the difference between temperatures and associate them back to the vertices of the 3D model. The color coding added through the jet color map takes advantage of the full color range by setting the minimum difference to the first row of the color map, the maximum difference to the last row and linearly interpolating the other colors in the temperature range. The result can be saved in a PLY format for further visualization in Meshlab.

### 2.9. Data Processing: Accuracy Assessment

The accuracy of the registration process can be visually and quantitatively assessed when examining the differential map. The temperature of the 3DTM remains (almost) constant during the post-walking and resting conditions, leading to an area with a differential temperature close to zero degrees on top of the 3D mesh of the 3DTM. This temperature difference should appear as a deep blue coloration. Any deviation from this coloration indicates a misalignment between the post-walking and resting maps on top of the 3D model. To determine the extent of this misalignment, the MeshLab measuring tool can be used to calculate the distances between the vertices of the 3DTM center and the color marks. The accuracy assessment on the differential map provides the full estimation of the workflow error as it is the sum of the errors in registering the post-walking and resting thermal images on the same 3D model. The approach provides a more comprehensive assessment of the accuracy of the full process compared to individual assessments of the post-walk and rest conditions.

### 2.10. Case Study

The feasibility of the workflow was tested in a case study involving a 65-year-old man with a traumatic third-proximal transtibial amputation. Based on the residual limb conformation, seven 2D thermal images for both the post-walk and rest phases were deemed clinically necessary for the description of the residual limb. During the walk phase, the subject was instructed to wear his prosthesis, which included a liner as an interface with the socket. The study was conducted in a controlled environment with monitored temperature and humidity levels of 23 °C and 58.5%, respectively. The temperature and humidity values were maintained within a narrow range of ±1 °C and ±5%, respectively, throughout the experiment, to obtain reliable and consistent measurements without compromising the well-being of the subject or introducing any unwanted external factors that could impact the results [32].

## 3. Results

### 3.1. Calibration

At the end of the thermal optics calibration process, the mean reprojection error was 0.32 pixels. The camera and lens distortion parameters are reported in Table 2.

This section may be divided by subheadings. It should provide a concise and precise description of the experimental results, their interpretation, as well as the experimental conclusions that can be drawn.

### 3.2. Thermal Images

**Figure 6:**
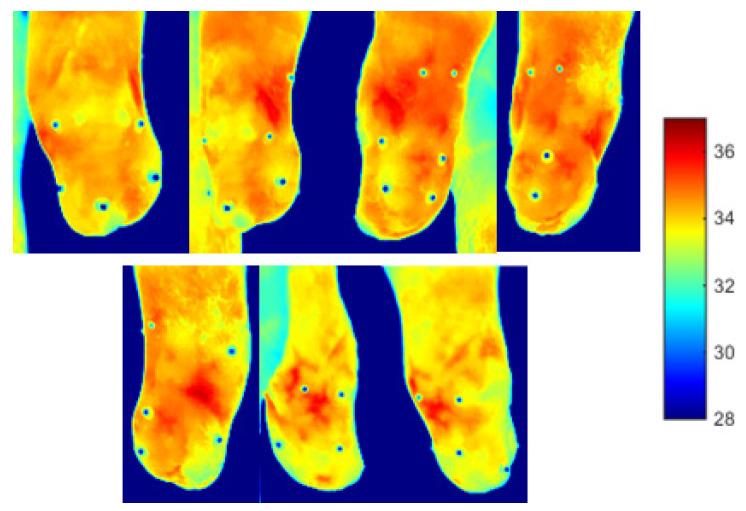
Thermal images obtained immediately after walk. The thermal markers are visible on the surface of the limb.

**Figure 7:**
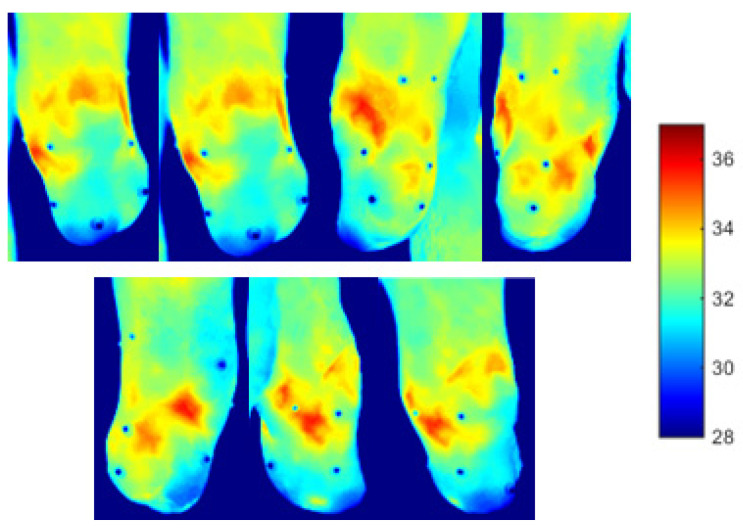
Thermal images obtained after 15 min of rest. The thermal markers are visible on the surface of the limb.

A two minutes acquisition process was required to collect seven 2D thermal images of the residual limb for the post-walking (Figure 6) and resting condition (Figure 7). Walking generated an overall heating of the limb over its entire surface. After 20 min of rest, the limb surface showed an overall reduction in the skin temperature. The distal portion of the limb reached the lowest values of skin temperature (about 30 °C).

### 3.3. Kinect Acquisition

**Figure 8:**
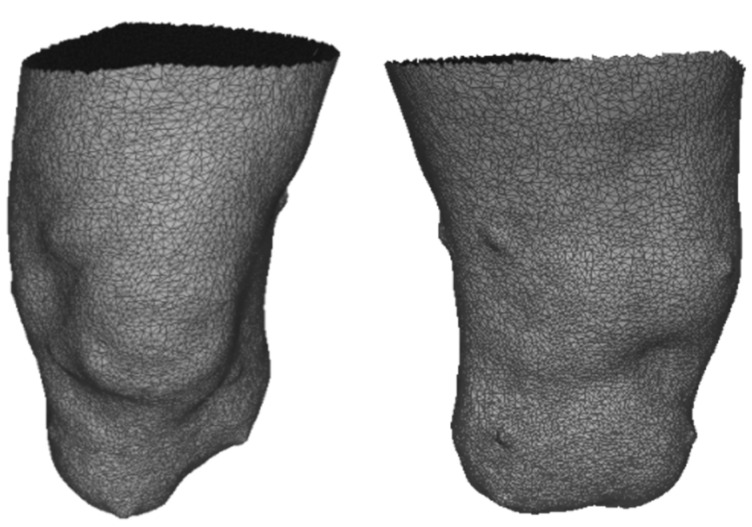
Anterior and superior views of the elaborated 3D surface of the limb. The markers are visible on the limb surface.

After less than 90 s of acquisition, a 3D surface of the scene with about 1 million vertices was obtained. After the extraction of the ROI and the elimination of all internal vertices of the residual limb surface and mesh refinement, a 3D model of the limb with about 16 thousand vertices was obtained (Figure 8).

### 3.4. Integration of the 3D Model and Thermal Images

**Figure 9:**
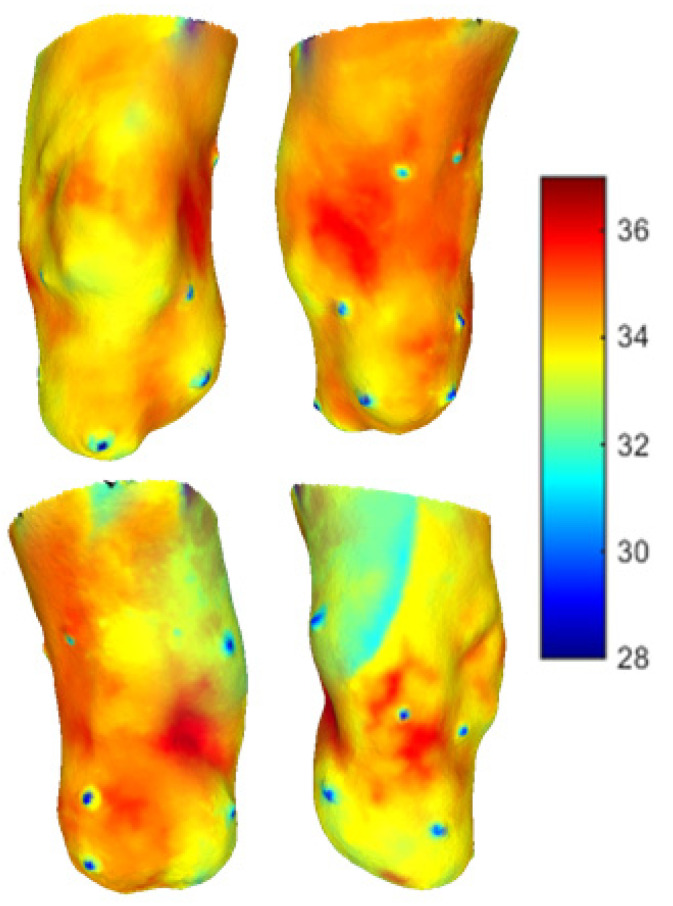
Post-walk 3D thermal map. Anterior, lateral, posterior and medial view.

**Figure 10:**
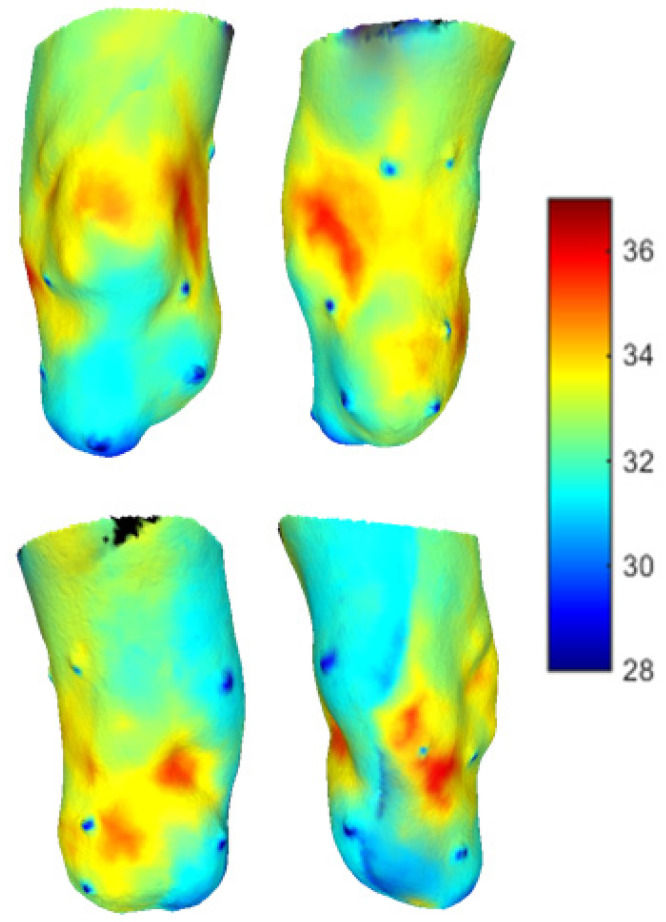
Basal 3D thermal map. Anterior, lateral, posterior and medial view.

After the integration process, the 3D thermal maps associated with the post-walking (Figure 9) and resting conditions (Figure 10) were obtained. Based on the results obtained from the analysis of 3D thermograms, the post-walking condition is characterized by regions exhibiting temperatures exceeding the baseline value, reaching average values close to 34 °C. Of particular interest are the suprapatellar area, two flaps and the popliteal fossa, which show temperature elevations reaching 36 °C. The basal condition shows a general drop in temperature, and the anterior apical area reaches temperatures of about 31/32 °C, but there is a permanence of four areas still at a high temperature (over 36 °C). 

### 3.5. 3D Differential Thermal Map

**Figure 11:**
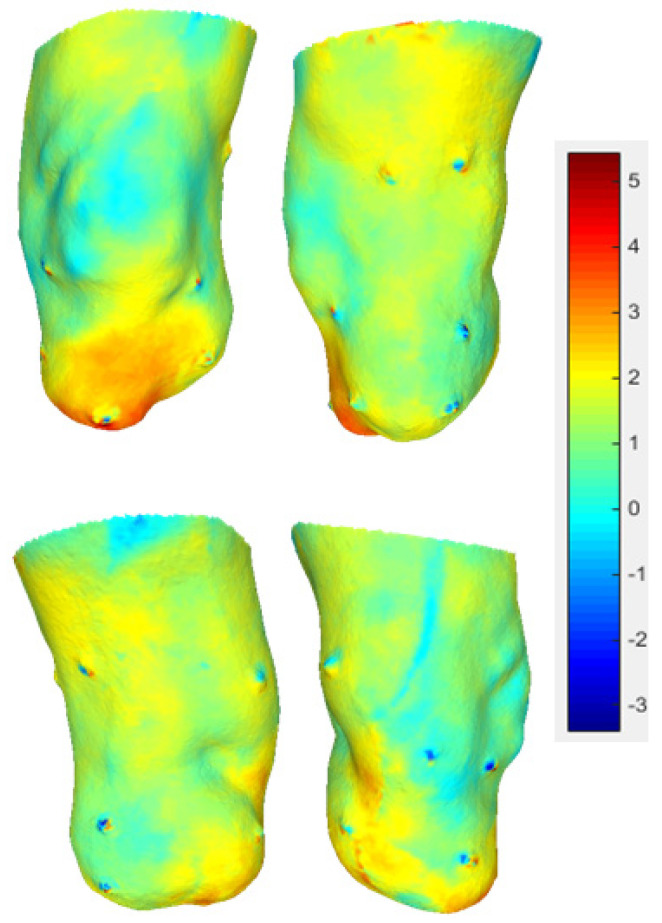
Differential maps: the areas that experienced the largest temperature variation (drop) are shown in red, while the areas that experienced the smallest temperature variation are showed in blue.

The differential map shows how 20 min of rest led to relevant cooling (3/4 °C) only in the anterior apical part, whereas the supra-patellar, flaps and popliteal fossa showed limited cooling of about 0–1 °C (Figure 11).

**Figure 12:**
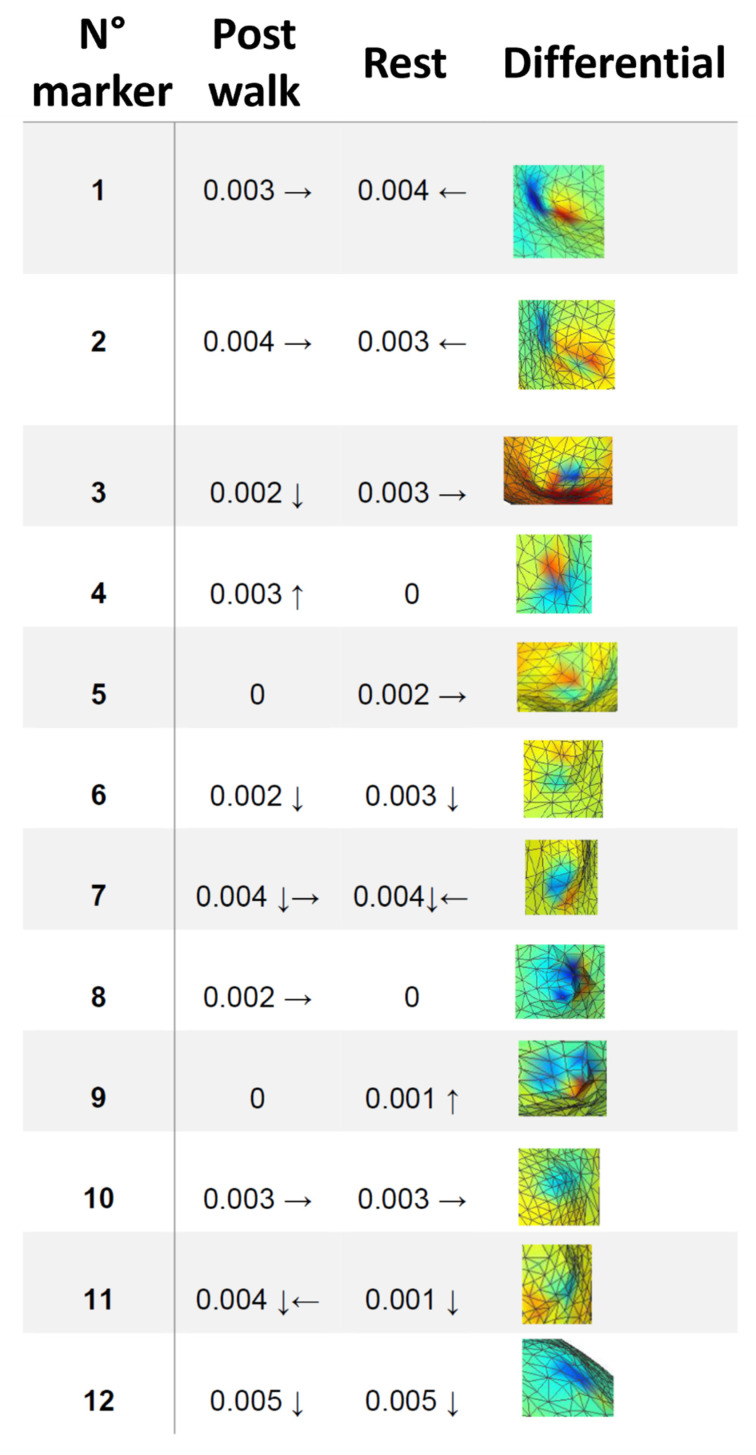
Misalignment in meters between the marker and the center of the blue halo (arrows indicate the direction of displacement).

In most cases, the areas around the markers present a red-blue stain, which represent the effect of the registration error (Figure 12). The median registration error was 2.7 mm (st. dev. = 1.6 mm, range = 0 ÷ 5.0 mm).

## 4. Discussion

The aim of the study was to advance an innovative workflow for the calculation of the 3D thermal maps of a residual limb of a person with transtibial amputation, e.g., to support the identification of areas of higher mechanical stress induced by socket use. Indeed, this information could be prospectively used to improve the fitting of prosthetic sockets when objective quantitative measures are needed to supplement uncertain or unavailable patient feedback or the limited experience of the patient or prosthetist.

In a previous work by [19], 2D thermal mapping was used to assess the most stressed points of the skin surface of the residual limb after level walking at normal speed for an individual wearing his own socket. Notably, even without pain or discomfort reported by the subject, the activity generated hot spots on the limb, which were potentially a source of skin lesions. In the present work, we found hot spots in almost the same locations around the knee joint, i.e., in correspondence with bone protuberances and/or skin-socket contact points, even after a rest of 15 min. Even in our case, the hot spots were painless and tolerated by the user. At present, the capacity for the prolonged stimulation of identical skin spots to induce medical complications over an extended period remains uncertain. Such an issue may be critical for subjects with diabetic amputation, in whom peripheral sensitivities are reduced by the degradation of vascularization induced by the pathology [33].

Thermal information was previously used in multiple medical applications, but details about the quality of the reconstruction are briefly reported. For example, the thermal information of the 3D reconstruction of a human head acquired with CT has been mapped in [22], with a mean registration error of 1.77 mm. We found a comparable registration error using a non-invasive technique. This was probably achieved thanks to the markers applied on the residual limb, which were interpreted by the software as irregularities to exploit for the registration. Due to their hollowness, the markers were valuable features for integrating thermal and geometric information. 

The registration errors registered on the differential map are clinically judged to be acceptable, since typical modifications during the fitting process use mechanical tools larger than the recorded errors. 

Noticeably, when compared to the 2D examination of multiple images, the availability of 3D maps substantially eases the interpretation of the results.

Although a variety of techniques exist to produce 3D models textured with thermographic information, the novelty of the proposed method can be summarized as follows: first, the technique was conceived to specifically address an open clinical issue with the fitting of protheses in lower limb amputees; second, the introduction of “thermal markers” supports the 2D−3D registration process and gives the intrinsic capability to monitor the registration error; third, the overall protocol is easy to replicate in clinical testing centers, thanks to the use of largely available free software. On this last regard, the ANN developed in MATLAB can be implemented without changes in Python-based alternatives. 

The major limitation of this study is the inclusion of a single subject. Indeed, our main aim was to propose a dedicated protocol and test its feasibility. Additional clinical trials are needed to test the reliability and reproducibility of the workflow, and to test its clinical effectiveness in terms of decision-making.

## 5. Conclusions

The present study demonstrated the feasibility of using a non-invasive and easy-to-apply algorithm for the registration of 2D thermal maps on the 3D surface of a residual limb, allowing us to obtain valuable information on the most stressed areas of the limb during prosthesis use. This information could be used prospectively for the manufacturing of prosthesis sockets. Our results also highlight the potential of thermal mapping as a tool for assessing the risk of skin lesions in individuals with amputation or other pathologies affecting peripheral sensitivity. Moreover, our findings suggest that the combination of thermal and geometric information could be exploited in several medical applications, paving the way for further investigations in this field. However, further studies are needed to evaluate the repeatability of the proposed method and its application to different anatomical districts.

## Figures and Tables

**Table 1:** Characteristics of the thermal camera.

Temperature Range [°C]	Image Resolution [pixels]	Accuracy [%]	Spectral Range [µm]	Thermal Sensitivity (NETD) [°C]
−20 ÷ 120	320 × 240	2	7.5 ÷ 13	<0.07@25

**Table 2:** Internal parameters of Kinect and thermal camera obtained after calibration and adopted for acquisition.

Camera intrinsic	Intrinsic matrix	[3 × 3 double]
	Focal length	[736.5414, 742.3704]
	Principal point	[274.4383, 145.2130]
	Skew	0
Lens distortion	Radial distortion	[−0.1004, −1.0318]
	Tangential distortion	[0, 0]

## Data Availability

Not applicable.
